# Profiling the Self in Mobile Online Dating Apps: a Serial Picture Analysis

**DOI:** 10.1007/s42087-021-00195-1

**Published:** 2021-02-13

**Authors:** Johanna Lisa Degen, Andrea Kleeberg-Niepage

**Affiliations:** European University of Flensburg, Flensburg, Germany

**Keywords:** Picture Analysis, Mobile online dating, Profiling the self, Profile picture, Types

## Abstract

Profiles in the widely used phenomenon of mobile online dating applications are characteristically reduced to condensed information mostly containing one or a few pictures. Thus, these picture(s) play a significant role for the decision-making processes and success, supposedly holding vital meaning for the subjects. While profile pictures in social media are omnipresent and some research has already focused on these pictures, especially selfies, there has been little attention with regards to the actual self-presentation when mobile online dating. In this paper, we show the results of a reconstructive serial analysis of 524 mobile online dating profile pictures investigating how subjects present themselves in the context of a mobile online dating app. This context is highly specific and characterized by continuous and dichotomous judgments by (unknown) others, unseen competition, and permanent validation of the self. Despite the conceivable multitude of possible self-presentations, our analysis led to eight clear types of self-presentation. Contemplating on subject’s good reasons for presenting the self as one of many and not as varied and unique when mobile online dating, we refer to the discourse of the private self (Gergen, The saturated self: Dilemmas of identity in contemporary life, Basic Books, New York, 1991; Rose, Governing the soul: Shaping of the private self, Free Association Books, London, 2006) and to (Holzkamp, 1983. Grundlagen der Psychologie. Frankfurt a.M.: Campus.) concept of restrictive and generalized agency in a context of socially constituted norms.

## Finding a Match Based on a Single Picture: the Characteristics of Online Mobile Dating

What once was a dress, a suit, the perfume, make up, the car to arrive in, shoes, body language—embodied knowledge and the first impression—is now captured in one picture when mobile online dating. Today, the dating reality of millions of users comes in the form of digital dating applications on mobile devices characterized by portability, availability, locatability, and multimediality (Schrock, 2015). These deliver (nonstop) algorithm-based suggestions of possible partners with the obvious effects of a multiplication of options and acceleration of the search for a partner, as well as a specific frame for the self-presentation, namely the individual profile picture. This is the current stage of a long history of changes in dating practices and the establishment of intimate relationships: once it was the arrangement of a marriage, suggested spouses by the social circle like family and friends, it then became more a question of coincidence in terms of, do I meet a spouse in everyday life? The former patterns were mostly local and social bound (Weigel, 2016)—one met who was in reach, a crucial characteristic also of former analogue matchmaking services. Online dating agencies then overcame the characteristic of the locally bound but sharing the characteristic of matchmaking based on rich and complex information. Online dating websites included extensive psychological questionnaires to pair the right and presumably matching individuals. Here the suggestion of a partner is detached from friends and family and their expertise, to the ‘expertise’ of shared lifestyle and onto a calculating tool, still striving to match on the basis of personal and detailed information about each individual. Matching partners due to professionalized websites, was the first digital example to overcome distance and to detach from both social surrounding, everyday practices and geographical position—one could find a partner on a national or even international basis broadening the dating pool. Today, however, dating is increasingly practiced as mobile online dating via applications, also known as local-bound-online- or microdating, with two main aspects that have changed significantly. First, the dating apps returned to locally bound principles fueled by GPS, suggesting a spouse geographically nearby and physically accessible. Second, the apps operate with limited condensed information, as only a minimum amount of information like age, sexual orientation and gender is required and decision making then is based on mainly one or a few pictures.

Based on such scarce information the app works as a matchmaker and expert, suggesting partners based on the following criteria: physical location, age, gender, and sexual orientation. Even though profile texts of up to 500 characters are possible, research shows that the decision whether to like or dislike a profile is mainly based on the impression of the first profile picture (Ward, 2016). Profile texts function mostly as an appendix and are often not considered or read at all (Degen & Kleeberg-Niepage, 2020). This lack of engagement with profile texts can be explained by the accelerated and dynamic practice of swiping along (ibd.). This does not deny a significance of the profile texts, yet the profile-pictures play a predominant and crucial role in the practice of mobile online dating. Profiles of proposed partners—typically the first picture allows the first impression of the suggested other—respectively give the other an impression of oneself. This might sound similar to analogue dating, like meeting in a club dancing, but unlike analogue settings, the picture is a static and scenic construction of the self. The presenter chooses motif, image section and perspective, selects one specific picture for the specific context. Moreover, the presenter has the options to edit, an intentional process of presenting the self in terms of optimization, success, and reacting to an (assumed) external feedback.

Despite the obvious importance of profile pictures in online dating apps and the general fact that pictures play a (more and more) important role in subjects’ lives and experiences, psychological research so far does not address the significance of (these) pictures, but largely ignores them as independent data (Reavey, 2016). To bridge this gap and to explore how subjects visually present the self in the context of mobile an online dating, we conducted a qualitative serial analysis of 542 Tinder profile pictures.

In this paper, we will first shortly review the state of research in the field of mobile online dating and in profile pictures and discuss significant theoretical perspectives to grasp the specifics of visually presenting the self in this context. Subsequently, we introduce the methodological framework and methodical procedure of our qualitative serial analyses of profile pictures. Then we present the typology that emerged from the analysis and discuss these results referring to (a) the discourse of the private self (Gergen, 1991; Rose, 2006) and (b) to Holzkamp’s (1983) concept of restrictive and generalized agency in a context of socially constituted norms. With the hypotheses derived from the serial analysis, we finally give an outlook on the upcoming single picture analyses and future research perspectives in general.

## Research on Mobile Online Dating and the Profile Pictures

There is a growing amount of interdisciplinary research of the social sciences on online dating applications. This research comes from different disciplines, most prominently the field of media and communication, with valuable perspectives (e.g., understanding a selfie in regard to sexual orientation, analyzing the development of codes and norms in communication, usage of emojicons and so forth, i.a. Highfield & Leaver, 2016; Duguay, 2016a, b; Courtois & Timmermans, 2018). Psychological research has so far focused on personal traits and behavior regarding the degree of honesty when presenting the self or on specific traits and personality characteristics of dating-app users (Charpenter & McEwan, 2016; Musil et al., 2017), user’s motivations (Timmermans & DeCaluwe, 2017; Ward, 2016) or the extent of disclosure (e.g., their face) (Fitzpatrick et al., 2015), accuracy of profile (-pictures) (Duguay, 2016a, b; Hancock & Toma, 2007) or potentially hidden information in the profile pictures in the form of conclusions about ones personality based on the presentation (Ward, 2016).

While there is some research about profile pictures (e.g., on Facebook), little research regards profile pictures in the specific context of mobile online dating. The little research deals with profile pictures in the context of mobile online dating specifically subdivides into qualitative and quantitative approaches and their respective perspectives.

Research following a quantitative, structural perspective demonstrates general behavior and the correlation of profiles and personal traits. Ranzini and Lutz (2017) combine location-based real-time dating and personal traits and picture analysis in regard to narcissism, loneliness, and self-esteem with motives and real or deceptive self-presentation. They show that realistic, that is more authentic, presentation tends to correlate positively with friendship-seeking motives and negatively with validation motives. Authenticity is higher when motives are relational and lower when having lower self-esteem, which also is correlated positively with sexual orientation. Sedgewick et al. (2017) found that the angle of selfies is gender related, men taking pictures from below and women from above. Another large quantitative analysis of 22 million online dating profile pictures show that younger men often show up in sporting activities, with animals or weapons and older men would display luxurious accessories like boats, motorbikes or champagne. Younger women, on the other hand, more frequently show off their bodies (e.g., by showing themselves in swimwear), place themselves in attractive or exotic landscapes, while older women would also display luxury goods like designer clothes (cf. Pleines et al., 2017). This research perspective mostly concerns assumptions about the intentions of the users which have been derived from the photographic representations whereby the photo has mainly been used as an illustration of (supposedly) inherent motifs (e.g., with wearing a swimsuit as a signal for the readiness for sex) (ibid).

The qualitative perspective mainly focuses on subjects meaning making, intentions and behavior when creating profiles. Ellison emphasizes the specific role of profile pictures when online dating: “Profiles are essential for online daters because they constitute a gateway for future FtF (face to face) dating” (2012, p. 2). She continues that it is specific for mobile online dating that decisions about a possible romantic partner are made within seconds, which leads to concerns about missing good options. The self-presentation furthermore is characterized as cue-reduced, as a process of selective, fragmentized and condensed self-presentation, where the information one gives about oneself is “malleable and subject to self-censorship” (Ellison, 2012, p. 3).

As mobile online dating is a process of asynchronous communication, it changes the general aspects of the presentation of the self, as it creates a time lag with possibilities to edit and modify, which also results in a tension between being attractive and honest (Walther, 1996, 2007). One can thoroughly create and optimize a profile and pictures, meaning the self-presentation is a planned enactment with a decisive time advantage, but this leaves the user with the question to what degree the self should be optimized to avoid insecurity and disappointment when meeting in real life. These characteristics result in plenty of research about honesty, deception, and authenticity and following rules and acceptance. Some degree of optimization seems collectively acceptable, for instance small optimization of professional status. In contrast, other deviations between analogue person and digital profile is highly condemned, for instance the body type or hair length (Degen & Kleeberg-Niepage, 2020). The acceptance of the (supposedly little) lies or rather optimizations are not always interpreted as lack of authenticity, when they are intentionally and realistically obtainable in the future, they are instead mutually accepted (Ellison et al., 2006).

Thus, the state of research only entails work on optimization and modification or raises questions about intentions and perceptions of self-presentation so far. A reconstructive perspective on series of profile pictures accessing the picture’s inherent logic has not been carried out. Such an approach can contribute to explicate socio-cultural storylines as frames of orientation, norms, and usual practices and thus the presenters’ often implicit knowledge on rules and standards of self-presentation in the context of online dating.

## The Self and Its Profile Picture

Images are omnipresent and constantly gain importance in digital realities, social media, profiles, and documentation of everyday practices (Reavey, 2016). In this context, the self-presentation of subjects becomes more and more important either as a means of recognizability (like a Facebook profile picture), or to tell stories about one’s life or current activities (Instagram, WhatsApp status).

For mobile online dating, profile pictures are even more important than in other social media because decisions of like or dislike are mainly based on one picture representing “it all” or rather “what’s recognizable and likable” (Ward, 2016).

In these digital dating contexts, subjects seek and receive feedback and evaluation regarding their visual self-representations. Although this might be similar to analogue encounters—as social feedback is a vital source for self-related cognitions and emotions (Gergen, 1991; James, 1890; Mummendey, 2006; Harter, 2012)—in digital contexts feedback and evaluation is only directed to a two-dimensional representation of the person, not to the person as a whole. Nevertheless, these judgments are experienced by the whole person influencing self-perception and self-esteem and thereby the way the self is presented in the future. As a kind of self-protection many social media profiles can be turned to a private-mode enabling users to select carefully who is able to see and comment on one’s pictures.

In mobile online dating, however, this possibility of self-protection is no longer there. Creating a profile means to open up to a wider public, to an assembly of unknown others, at least within the range of the predefined criteria. Furthermore, the context of online dating with its aims of finding an intimate partner—for either one night or for the rest of one’s life—leaves subjects in an especially vulnerable position with negative evaluation resulting in a threat to the self. Additionally, compared to other social media, negative evaluation within mobile online dating apps is rather implicit for one will never know how many people looked at one’s profile without liking it and profiles cannot simply be commented on negatively. Therefore, knowledge about one’s success or failure can only be acquired by comparing one’s likes, matches and dates with others (e.g., friends or colleagues) developing a competitive logic.

Of course, comparison and competition with others is an inevitable source of self-related cognitions, emotions and behavior, of self-confidence, self-perception, and self-esteem (Festinger, 1954; Morse & Gergen, 1970) with positive evaluation pushing and negative feedback decreasing it. Nevertheless, the self-presentation in an online dating profile, being reduced from a complex process of verbal, para- and nonverbal behavior and activities (Goffman, 1959) to a few static characteristics, leaves the subject with limited options to prevent the self from setbacks. When one comes off worse in such a competition (i.e., having few matches or dates), subjects—besides optionally leaving the app or lowering their expectations—change and thereby supposedly optimize the profile (picture) to increase success. For the optimization subjects then occasionally reach out for help by friends or colleagues and preferable those being more successful, copying profile styles and texts or even turn to professional tutorials and coaching (Illouz, 2007). Such optimization of one’s self-presentation is reminiscent of what Rose (2006) and Gergen (1991) call the discourse of the private self with subjects taking responsibility for previous failures, internalizing them into the self.

However, for one’s self-presentation subjects must largely refer to an unknown and instead imagined audience with few cues to rely on (Goffman, 1959, 1981; Litt, 2012). To please this imagined audience users have to relate to—often rather implicit—norms of self-presentation they expect to be successful in an online dating context. When relating to these norms, subjects have at least two options: one option would be to follow and submit to the abovementioned competitive logic of success and adapt one’s self-presentation accordingly, exercising what Holzkamp (1983) called restrictive agency. A second option would be to avoid a quantitative logic of comparison and competition or even consciously reject competition at all, creating a highly specific profile and e.g. emphasize other criteria of success like the intensiveness of the dates. Such activities would provide a more sovereign subject position and maybe come close to what Holzkamp (ibid.) called generalized agency.

These theoretical considerations provide the framework for our serial analysis of profile pictures and for responding to the following questions:

How do subjects shape—construct and reconstruct—the self in the specific context of dating app profiles (accelerated daily practice, continuous judgment by others, unseen competition, validation of the self, exposure, self-esteem), in the tension between authenticity, exposure, and subjectivity on the one hand and the implied social norms and disclosure to unknown others on the other hand? How do subjects position themselves towards these norms in terms of subjective sovereignty, conformity, and moments of resistance?

## Methodological Framework and Methodical Procedure

### A Reconstructive Perspective on Pictures as Social Practice

Our research question is aimed at the meaning making processes involved when subjects visually present the self in a mobile online dating app profile. This focus and the fact, that an analysis of such pictures is a still novel and rather explorative endeavor calls for a qualitative procedure which is targeted at a deeper understanding of subjects’ sense-making procedures and the development of new presumptions. Reconstructive methods follow specific quality criteria—transparency, intersubjectivity, scope—that differ from those of quantitative research (Steinke, 2010). These are based on the epistemological assumption that reality does not exist independently of the observer but is constructed in a creative and reciprocal process between empirical data and researcher. Because of these particularities, replication would be neither possible nor useful; we therefore place particular emphasis on the transparency of our approach in the following sections.

Instead of asking people for their interpretation of the pictures and thereby producing a narration about pictures, we have analyzed the pictures as such. In doing this, we consider pictures as social practice and interactive behavior (Pilarczyk & Mietzner, 2008) and as expression of subjects’ (often implicit) knowledge of the social world and their own position in this world. We therefore assume, that (an analysis of) pictures will provide access to people’s ways of relating the self to this world and thereby their (perceived) scope of action.

Such an understanding of pictures corresponds to the reconstructive research paradigm within which specifically Documentary Method developed a picture-analytical procedure to reveal the creators implicit (or atheoretical) knowledge contained in the pictures (e.g., Bohnsack, 2010; Bohnsack et al., 2015). Nevertheless, picture analyses within the reconstructive research paradigm almost exclusively focus on single pictures or on small series (e.g., Michel, 2015) whereas larger samples or series of pictures are rarely addressed.

For our purpose, we relied on and combined the only two approaches (at least to our knowledge) that are reconstructive and dedicated to large datasets—serial analysis by Mietzner and Pilarczyk (2008) and first impression analysis (Ersteindrucksanalyse) by Müller-Dohm (1997). Both differ in their procedure and the ascribed meaning of the serial analysis for the research project. For Müller-Dohm, first impression analysis is a preliminary step that precedes the actual detailed case analysis: “In order to achieve a first systematization of the […] material, it is extremely helpful to carry out so-called first impression analyses of all cases prior to the actual individual case analysis” (p. 102, translated by the authors). In contrast, for Mietzner and Pilarczyk both serial and single picture analysis are equally significant and do not have a strict order: “The aim of serial analyses is to detect continuous or discontinuous developments, anomalies and differences as well as to classify and interpret them.” (p. 142, translated by the authors). Such an analysis “offers the chance to recognize and interpret the enforcement of image patterns and styles” (ibid.) and enables to understand and detect specifics, which is impossible when exclusively studying single cases.

Despite these differences both approaches share with Documentary Method the reference to art-historical procedures, especially to the work of Erwin Panofsky (1955), who at his lifetime changed art-historical picture analysis fundamentally, namely from iconography to iconology. Panofsky understood a picture as a document of an epoch or of the personality of the artist and developed a three-step method to access the picture’s inner structure. His three-step out of pre-iconographic denotation, iconographic interpretation, and iconological analysis is often described as the shift in analytical attitude from the what to the how (Bohnsack, 2010).

Panofsky's (1975) approach and methodological steps were demonstrated by a simple scene, the pulling of hats, when two people (in the early twentieth century) meet on the street.

Step one (pre-iconographic denotation) of Panofsky’s iconology describes the strictly formal appearance that excludes all interpretative knowledge and assumptions as well as cultural meanings. In our scene, this corresponds to the statement of a gesture—a person lifts his hat as he passes.

Step two (iconographic interpretation) approaches the meaning of the scene by assuming so-called um-zu motifs (Bohnsack, 2009, p. 30), in our scene, for example: A gentleman lifts his hat to greet.

Step three (iconological analysis) opens up the historical and socio-cultural meaning of the scene and considers the gesture of pulling one's hat as a class-, gender-, culture-specific practice. This is understood by the other only if he shares his space of experience.

By means of this procedure, an image can be grasped in detail and with the bracketing of one's own prior knowledge, and analyzed for all possible meanings in the respective cultural context.

### Data Collection and Analytical Procedure

#### Data Collection and Sample

We collected profile pictures, a total of 542 heterosexual male and female profiles which we gathered via two specially created heterosexual profiles (one female, one male) in a northern German city. We chose Tinder as one example of a local bound mobile dating application for two main reasons. First, it is currently the most popular application in Germany and worldwide with 56 million users (Statista, 2020; Ward, 2016). Second, earlier research showed that, specifically in this application, the pictures play a significant role when deciding on possible partners, with other details such as the profile text playing a minor role. We collected the data on a day in November 2017 between 6 and 11 pm (242 profiles) and a day in May 2020 (same time and same location with 300 profiles). The targeted age group was 25–33 years old. We switched between male and female accounts every half hour to make the sample as equal as possible regarding external impact.

#### Procedure of the Serial Picture Analysis

For the concrete procedure of analyzing the whole series Müller-Dohm (1997) suggests three steps. First, the description of the first impression of the picture, i.e. the primary message, objects and persons that are depicted, stylistic elements, and primary presentation; second, the preliminary formation of types based on similarities; and third, the formation of types including the selection of a prototype. Mietzner and Pilarczyk (2008) who consider a sample of 300 to 500 pictures as suitable for a serial analysis propose different strategies like arranging series of motives to register changes (e.g., over time, to record the elements of the picture design, common motives or topics) to eventually create types as well. While for Müller-Dohm the term 'type' denotes similarities on the level of motifs, Mietzner and Pilarczyk go beyond this and ask about the socio-historical significance of frequently appearing themes, motifs, and modes of representation (ibid. p. 143f). For our analysis, we combined these suggested procedures while conducting a three-step procedure as well.

1. As a first step, we split into two groups and described the comprehensive samples motifs, strictly descriptive to get an understanding of the profile presentation.

We described the profiles regarding the person visualized in terms of perspective/angle/body parts, gaze, gesture, posture, mimic, dressing, objects used, background, distance from camera, background, items, and other subjects and compared the results. This procedure is close to the first step suggested by Müller-Dohm (1997) and corresponds to Panofsky’s (1955) pre-iconographic description.

Example: Subject/person, full body posture, showing the face including the eyes, palm tree in the background, wearing a light/short dress and a hat with a prominent badge on the front and a backpack—no requisites, no other people, no animals.

General sample description: The comprehensive sample shows that self-presentation on Tinder is dominated by images arranged around one person in the images’ center. The self-presentation can differ in terms of angles, backgrounds and props or objects, variation and degree of filter, yet there are some overall characteristics for certain kinds of pictures.

First there are motives with the person predominantly filling the image, displaying mostly the face in a close up, possibly displaying the bust or torso, with hair framing the face, sometimes little gestures with the hands or small objects like jewelry, glasses or pets, only little background insights without explicit indications for actions or interests are given. With this type, the background remains a secondary motive with little information. Typical backgrounds are nature surroundings, urban locations or interior/indoor locations.

The second general class of pictures displays the whole body/posture often supplemented by a background playing a larger role for the scene created. Here the background is part of a setting, often the person is using the background to demonstrate an action, interest or lifestyle and (also larger) objects like sports equipment are presented. Backgrounds show travel scenery and exotic landscapes or animals, specific surroundings like sports facilities, harbors or airports, urban architecture or statues and art in the form of street art or sculptures, bars/restaurants or clubs. These pictures can also contain other people, showing one other person or the subject among several others, posing for the picture or while engaged in an activity.

The third kind of self-presentation comes as pictures only showing parts of the body or the face, or the back of a person, or display objects or props to stay incognito like helmets, sunglasses, or umbrellas. Some single examples show no person at all but objects or avatars.

2. Based on a random sample of 140 pictures, we then arranged, rearranged, and compared the pictures following similarities and discrepancies according to Mietzner and Pilarczyk (2008), forming a kind of preliminary typology (according to Müller-Dohms second step, 1997). As we referred to common sense knowledge and external sources during this step, it corresponds to Panofsky’s (1955) iconographic interpretation.

Examples:

- Complete image of a young woman presenting herself against the background of an exotic landscape in summer clothing plus a hat with an Australian flag—refers to summer temperatures and stay abroad (often associated with vacation, leisure, travel, far from everyday life).
- Representation of a male subject in a beach scenery, wearing a wetsuit, and holding a surfboard. Displaying a situation of leisure activity, hobbies in a non-everyday practices’ environment, possibly holidays.

Both descriptions include information about activity, habits and lifestyle or taste and are in the final typology collected under the type “informative.”

3. With the last step, we confronted our preliminary typology with the comprehensive sample, extended or modified it where necessary and eventually developed our typology (based on Müller-Dohm, 1997 and Mietzner & Pilarczyk, 2008).

Saturation was reached with less than two hundred pictures. The great majority fit clearly into the typology; this also counts for the sample from 2020. We contemplate on the meaning of each type and subsequently situate the final typology within theoretical and empirical insights and discuss the meaning of our results for both subjects’ perspectives and their behavior with regards to social norms. This step parallels Panofsky’s (1955) iconological analysis.

## The Tinder Typology of Self-presentation: Being One of Many—countless Opportunities, Few Types

Our reconstructive serial picture analysis resulted in a typology with eight types of different magnitude (Table 1).

**Table 1:**
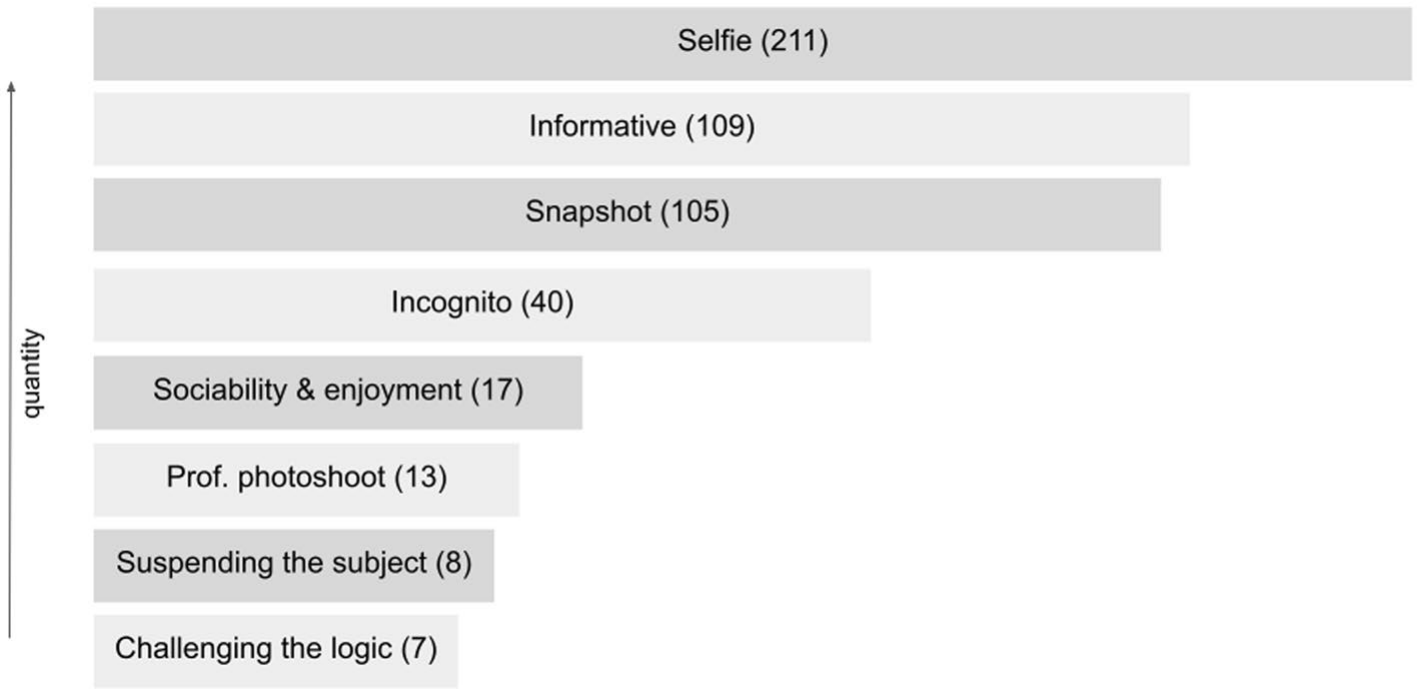
Tinder typology of self presentation (515 plus 9 COVID-19 mask)

Nevertheless, a bigger seize of a type does not necessarily equate to greater significance which can be seen from the following detailed presentation of the individual types. Please note: For anonymity, the picture depicted are reduced to a sketch and blurred in regard to background and details and furthermore censored in regard to the eyes and parts of the face, age and nickname. Thus, we assume that no harm for the user can occur from our publication (for further ethical considerations see “Ethics and Limitations” section).

The selfie type, characterized by self-control and limits:


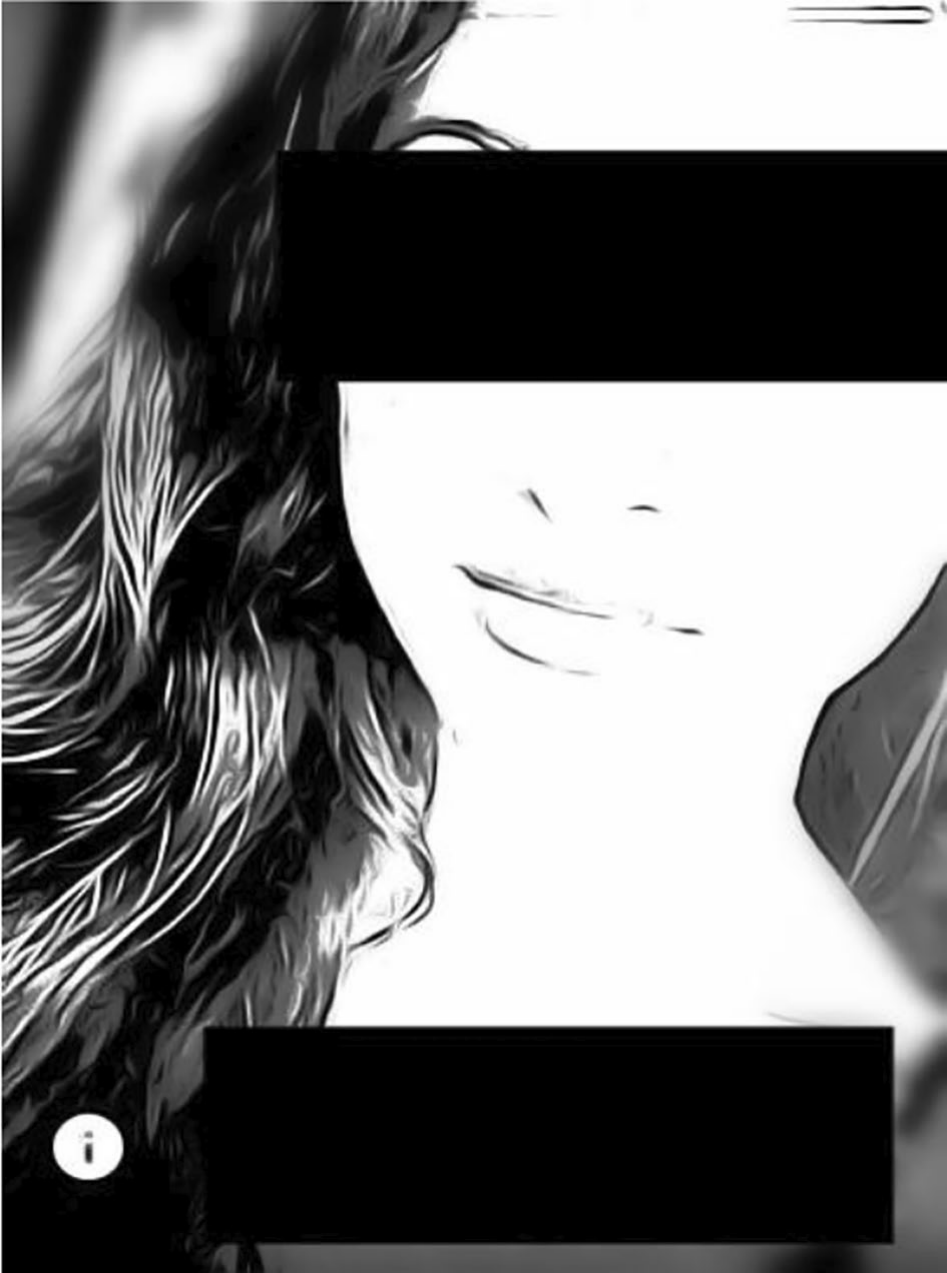
Description: A selfie is a picture taken by the person him- or herself where the angle, the proportion of head, shoulder, and arm show that photographer and photographed person are the same subject. While not hiding the fact of taking the picture him- or herself differences occur in posing, angle, person-camera relation, objects and gestures. There are different kinds of selfies coming with “direct eye contact,” where the subject looks into the camera,” “indirect eye contact,” where the person is watching the self on the screen, “showing skin” where subjects are (seemingly) partly naked, “no eye contact” where subjects seemingly look away or have closed eyes, look towards the ground, and “mirror selfies,” where the picture is taken in front of a mirror often showing the device and selfie “with context reference,” where the picture contains explicit information about the surrounding, for instance one person taking a selfie with an elephant.

Meaning: Taking a selfie is a moment and an act with the self. It makes obsolete the seemingly natural difference of photographer and photographed. On the one hand, the selfie is an act of control and full subjective agency; on the other hand, the act and the picture are restricting the objective to armlength and angle. This means the resulting picture is including the typical and explicit characteristics, e.g. showing the prominent shoulder. For subjects, taking a selfie has some advantages. First and foremost, they are controlling the moment of taking the photograph. Second compared to pictures taken from other, it secures the privacy of not socially exposing the enaction of looking good and ‘wanting’ a carefully orchestrated close-up and often multiple attempts striving for the perfect scene, angle, light and so forth—characteristics often perceived as unpleasant or awkward. Furthermore, it comes with the advantage of immediate availability and the spontaneous capturing of a moment where the person felt well or even happy or wants to capture a situation.

The informative type, characterized by lifestyle and romantic echoes:


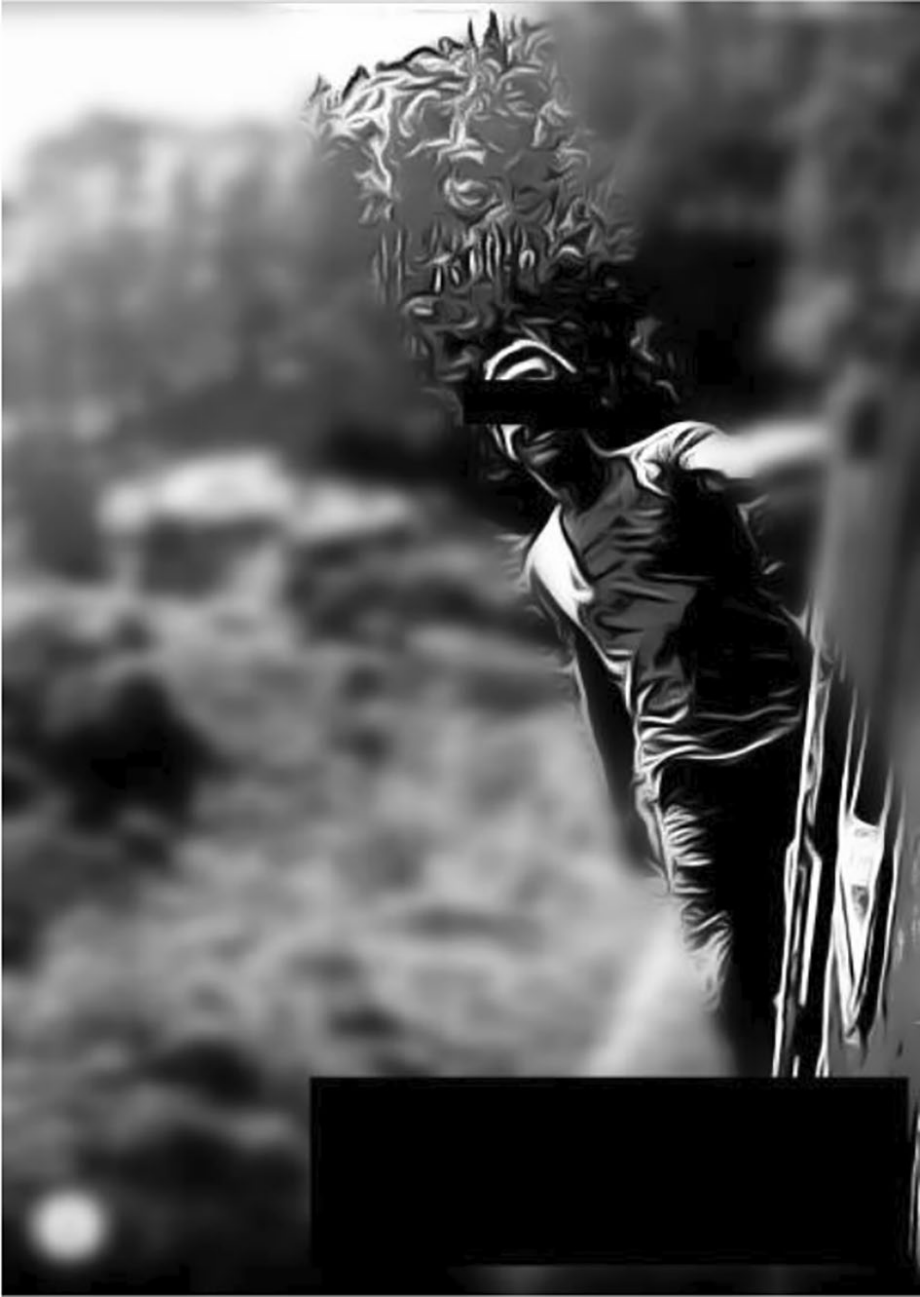
Description: Pictures of this type give insights into hobbies/activities/lifestyle by showing them in the background. The person here is either posing with for instance, sports equipment, in front of travel sceneries, when commuting, with a pet or an animal or in surroundings, for instance, a yacht club for eating dinner, golf course eco-friendly/fair-trade coffee places, or props and objects for sexual practices like whips or costumes (e.g., cosplay).

Meaning: The informative picture goes beyond the selfie in several ways. It is socially interactional because someone else is taking the photograph. At the same time, including cues from the background the motif leads to some distance between photographer and photographed person, sometimes the photographed persons face is hard to recognize. Even though it centers one subject, it gives insights/information about the background, possible hobbies, interests, an activity or specific experience or skills/capabilities and is thus hinting to a certain lifestyle. This can function as a correspondence to the romantic ideal of relationships, which contains aspects of exotic contexts, specialties, and consumption. These self-presentations invite the viewer not only to see the subjects face but function as an invitation to a possible lifestyle, a certain scenery to join, an idea of how it might be to be with the person and the course of life of an possible future. These pictures are presenting some extraordinary activity, the opposite of everyday life and responsibilities, profiling the subject as active, exceptional, international and with stamina. The presentation is an invitation to personal information or to a possible lifestyle and personal capabilities.

The snapshot type, characterized by a situated moment:


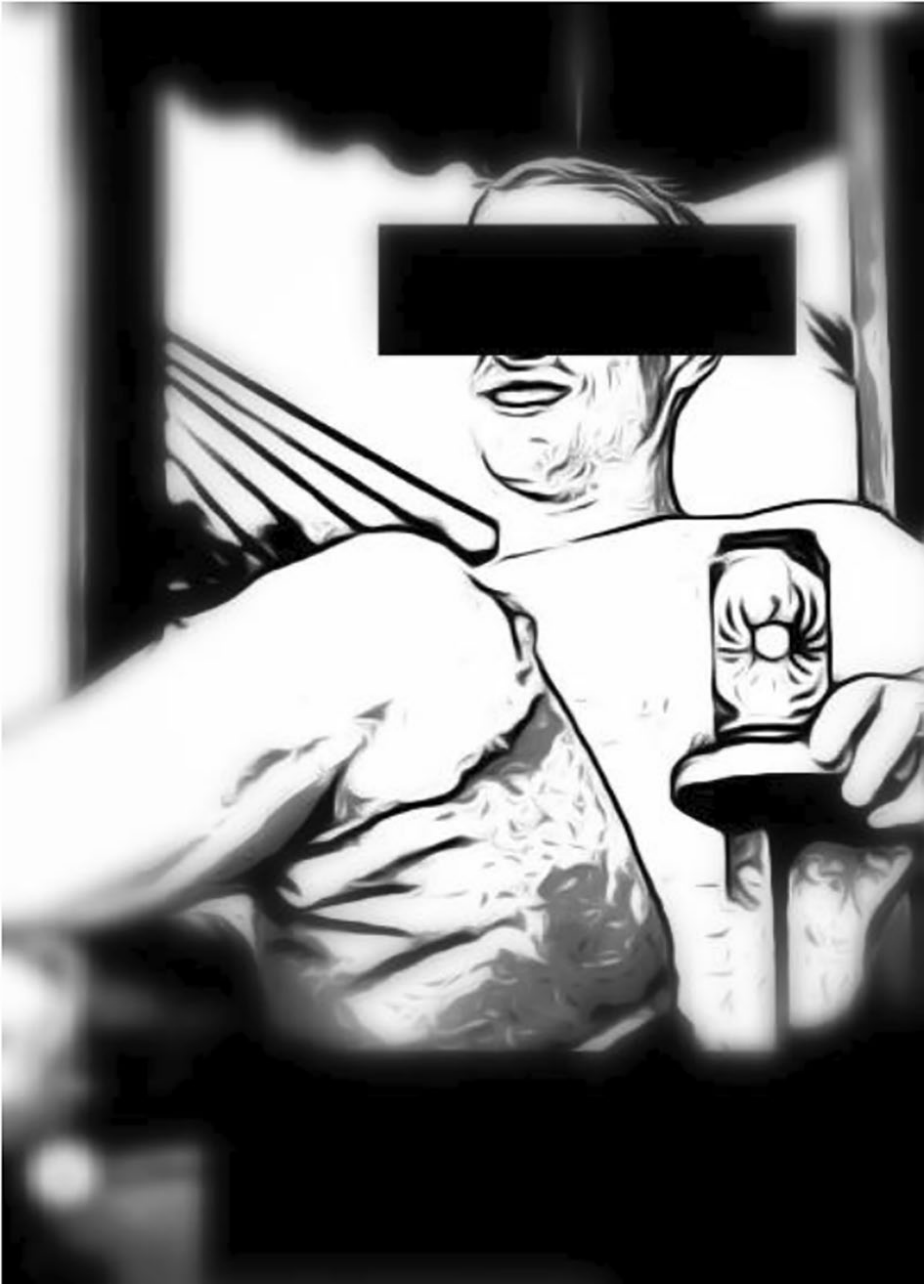
Description: Snapshots are pictures that seem to be taken without the photographed person being conscious of it, one is presented in a seemingly natural situation or engaged in an activity, e.g. giving a speech, walking a street and shopping, doing sports, simply being in action and explicitly distracted from the situation of being photographed. There occur several forms of presentation, the portrait, where the subject is close to the camera and the image shows facial features and facial expressions and only the torso is photographed. Further forms are “in motion” where the person is walking, cooking, or playing a game and literally moving in the moment the picture is taken and “posing,” which does not address posing for the camera but more posing in a context, often these are males holding a beer and a cigarette, legs spread in an urban scenery.


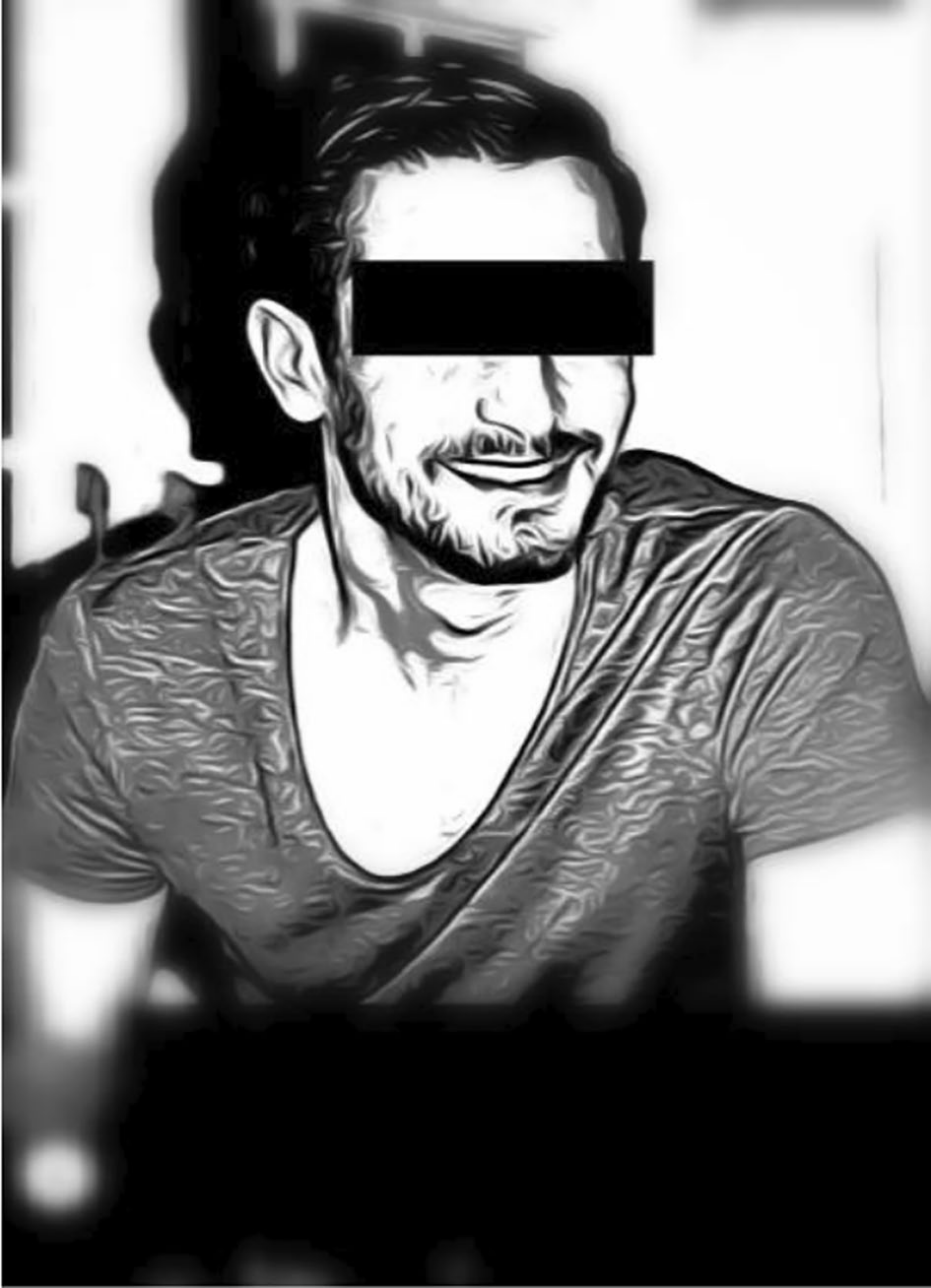
Meaning: The subject is in the center of the picture, where the face and facial (micro-) expression is recognizable and takes the audience into the atmosphere of the moment capturing and communicating the aura of the photographed subject. The distance is generally not as close as the selfie but closer than the informative and social types, and the context plays a minor role. The photograph is created in two subjects’ social interaction, whereby the photographed subject seemingly does not notice the picture being taken in that moment or at least they are not explicitly posing, it thus references a form of authenticity. The photographer is capturing a specific moment, where the subjects seem to have a moment worthy of being captured. This implicitly points to the social relation where the photographer is willing and focused on capturing a moment centering the other; thus, these pictures contrast with the selfie.

The type of sociability and enjoyment, characterized by cues to network and milieu:


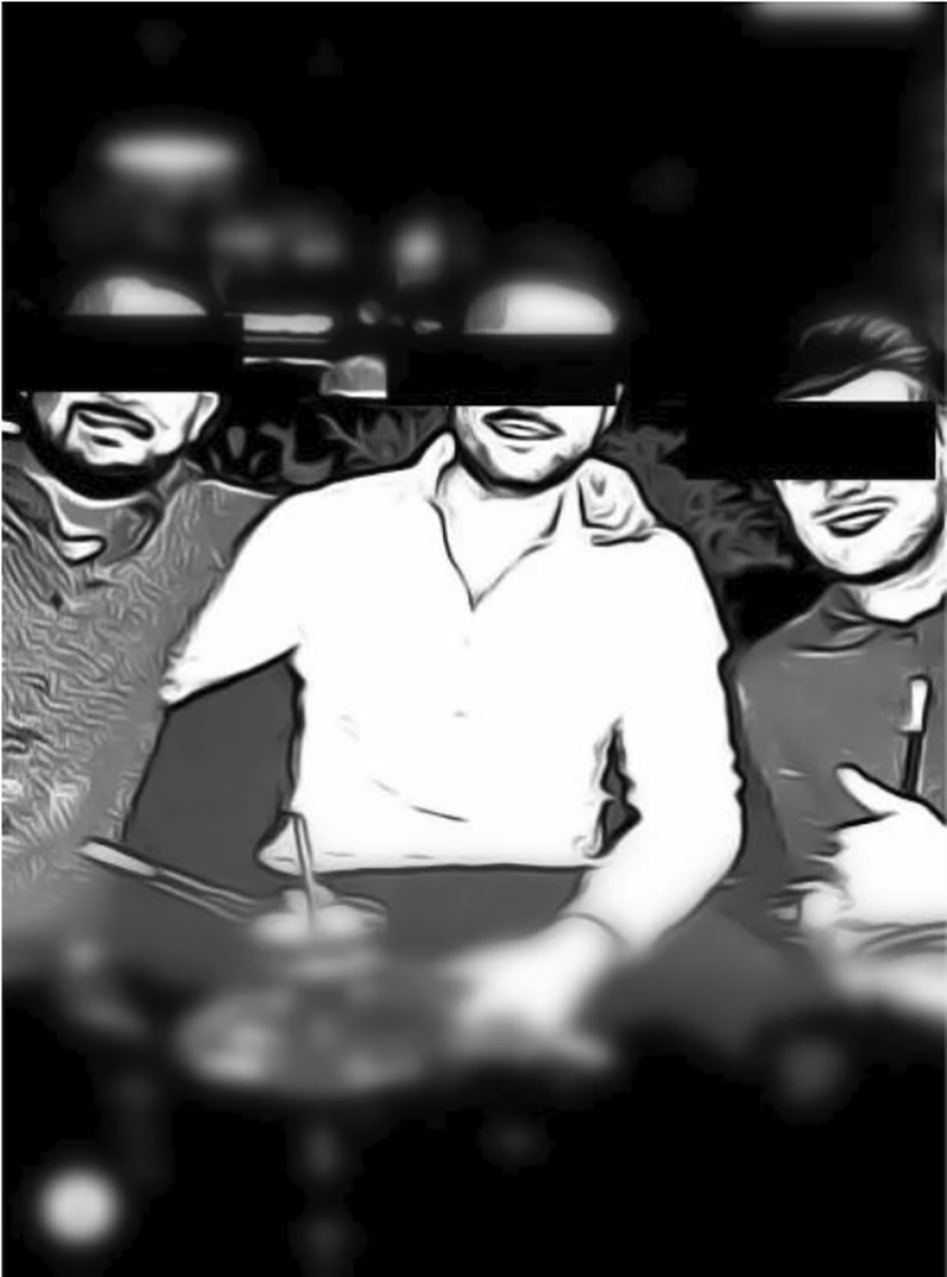
Description: This type shows one central person in a surrounding with other subjects, either as a group interacting or in a social context for instance a club in the form of a “party.” Often these images are arranged in context of food and drinks, smoking equipment, costumes and ceremonies like weddings.

Meaning: In comparison to the other types here there is more than one subject centered, often it is unclear which one is the profiles owner. These pictures also indicate some background, taste and activity, but moreover point to sociality, possible social competence, a lack of loneliness and profiles the subject as entertaining and as being a person worth being around. This presentation hints to a lifestyle of being social and around others, localizing the subject as being part of a bigger network/group and social contexts. This context, in some examples, references social responsibility in the form of children or elderly. These pictures are similar to the informative type giving insights into lifestyle and a certain course of life as a possible partner. In particular, this type gives insights into certain tastes and milieu, often these pictures are appear quite spontaneous, with little arrangement and (perhaps unintendingly) reflect places and interests (in the form of for instance sky bars, expensive hotels and clubs or shisha bars or a certain style of vacation).

The type professional photoshoot, characterized by an orchestrated self:


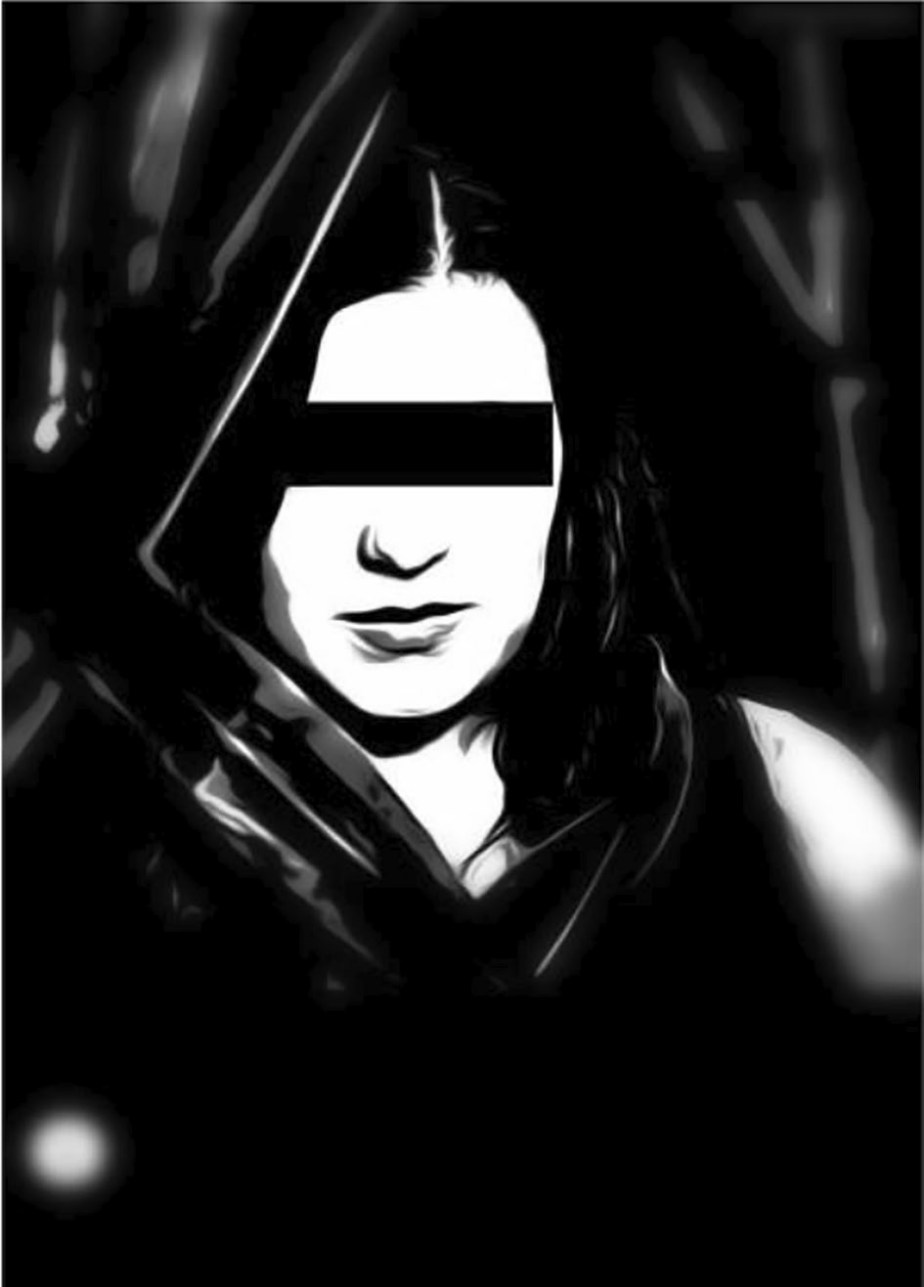
Description: These are pictures that seem to be taken by a professional photographer, meaning there is a clearly arranged set, lighting, and a high-quality image. A person here is posing for the picture. A specific way of presentation is the “CV/Resume picture”, which looks like a picture usually used for job applications or a LinkedIn profile, often the person is dressed quite formally (wearing a suit or a blouse).

Meaning: These types are special in terms of the photographer and photographed relationship as it is grounded in a an often paid and one-sided service. The centering of the photographed subject is based on this specific contract not on a social bond or intimacy, but the opposite as the photographer in most cases is a stranger conducting her or his profession as a service. The assignment is to focus on a specific look or feature (beauty, erotic, fitness, job application and so forth), thus focusing on themes and enhancing the emphasized characteristics and communicating the photographer’s general style as an artist, then the specific photographed subject. Furthermore, the situation is characteristically set-in scene/orchestrated by posing, light installation, and prerequisites such as specific clothes. The logic of the professional photograph is counterintuitive to the non-committal accelerated logic of low investment predominantly seen when mobile online dating as it is an explicit investment.

The incognito type, characterizing a hide and seek:


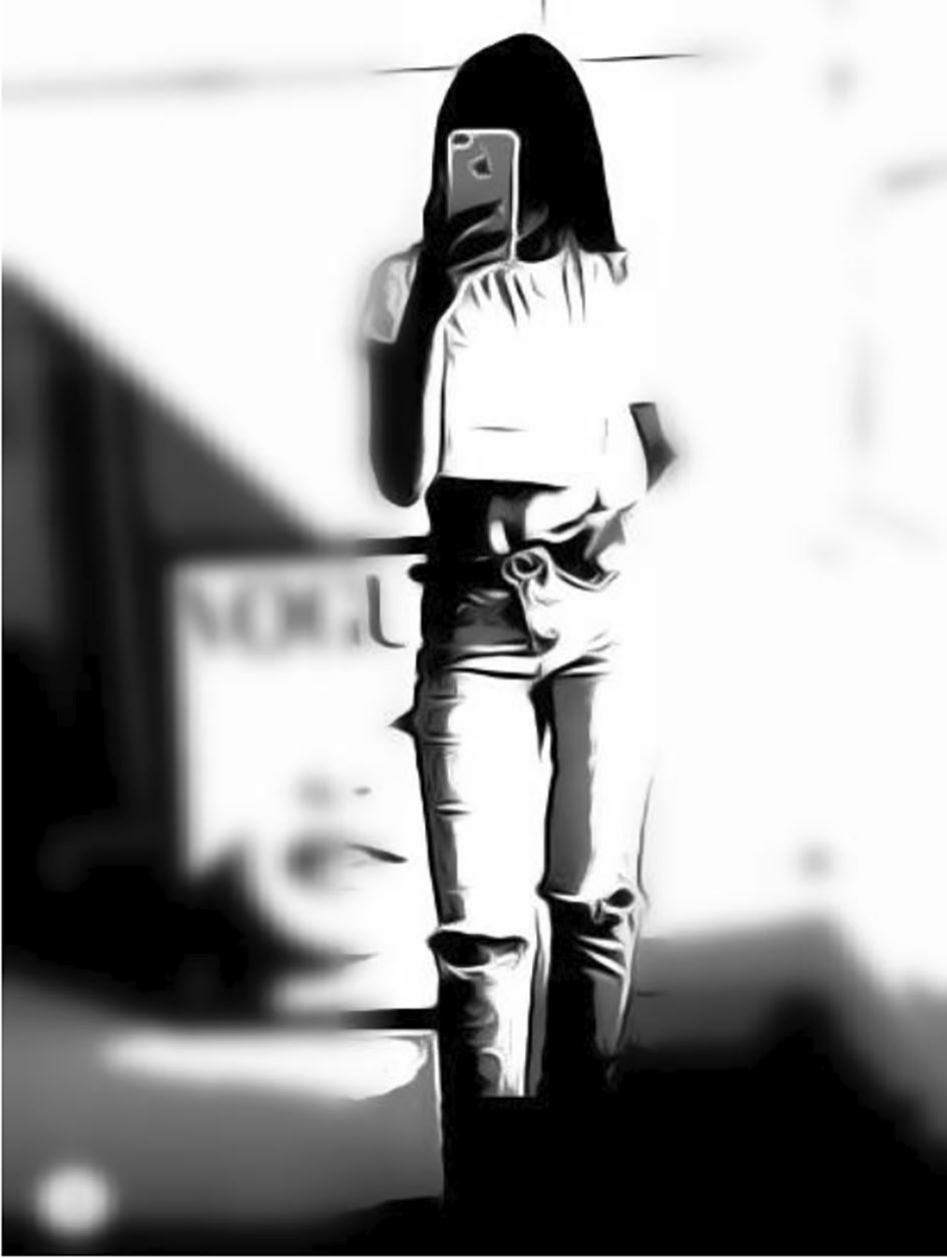
Description: This type of picture shows a person who is hiding or not showing their face. Either with an object like a hat, the cellphone, a hand, or an umbrella in front of the face, by showing the back, by only showing body parts or parts of the face or are taken from a distance that the subject is unrecognizable. Other present a tilted head or, mostly women, cover their face with hair either because the wind blows or demonstratively use it to cover the face.

Meaning: This type contains pictures of mirror selfies and selfies but also pictures taken from others, for instance where the subject is photographed from behind. The main characteristic is that the persons face is hidden and thus the subject stays incognito. Hiding here has many facets, taking mirror selfies hiding behind the device, covering the face with one hand, jewelry, a hat or with their own hair or choosing a picture taken from behind. Often these photos relocate the focus from the face or the whole person to the body or body parts like the lips or eyes and so forth. Being incognito and protecting the identity has meaning on several levels. First, judgment by the unknown other is relativized. Second, the logic of being presented and available is countered. Instead, the incognito picture reveals a political, normative or social stand and creates an artificial hurdle and exclusivity. The other has to dare the like, invest in the relationship and qualify for the reveal of the subject in terms of identity and look. This pattern constructs a sphere of mystique, a tension of the play with curiosity, control and what might hide behind the mask.

The type suspending the subject, characterized by seduction to projection:


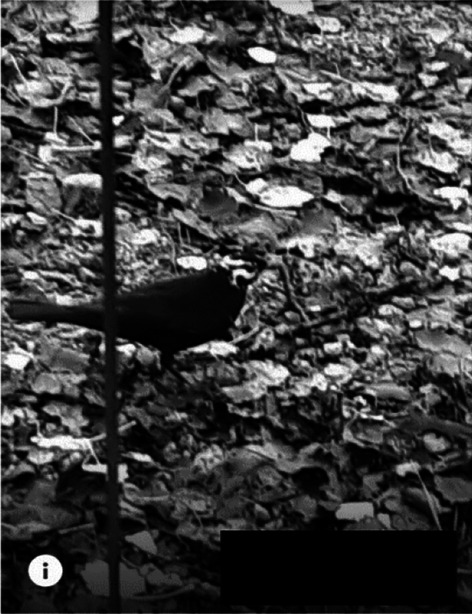
Description: A small number of pictures could not be classified following the typology shown above. These all hide the identity and furthermore the body of the account holder and instead an avatar, objects or animals are shown. One picture showed a fictitious character from a comic, two pictures showed objects like trash, one showed a bird, one a single flower. 

Meaning: The suspension of the subject is countering the logic of quick decisions based on seeing the possible partner. Similar to the type incognito the subjects create a (even stronger) sphere of hiding, curiosity, the dare to like at a possible future reveal. Here, there are no hints to the subjects looks, body type or features and furthermore the presented objects invite a huge range of interpretation, speculation, and projection—what does the audience understand from seeing a bird or trash? Is it saying something about the platform, the other or the self or neither of those? Leaving the other with the responsibility of what is seen and the decision whether to like or not.

Type: Challenging the logic, characterized by being counter habitual:


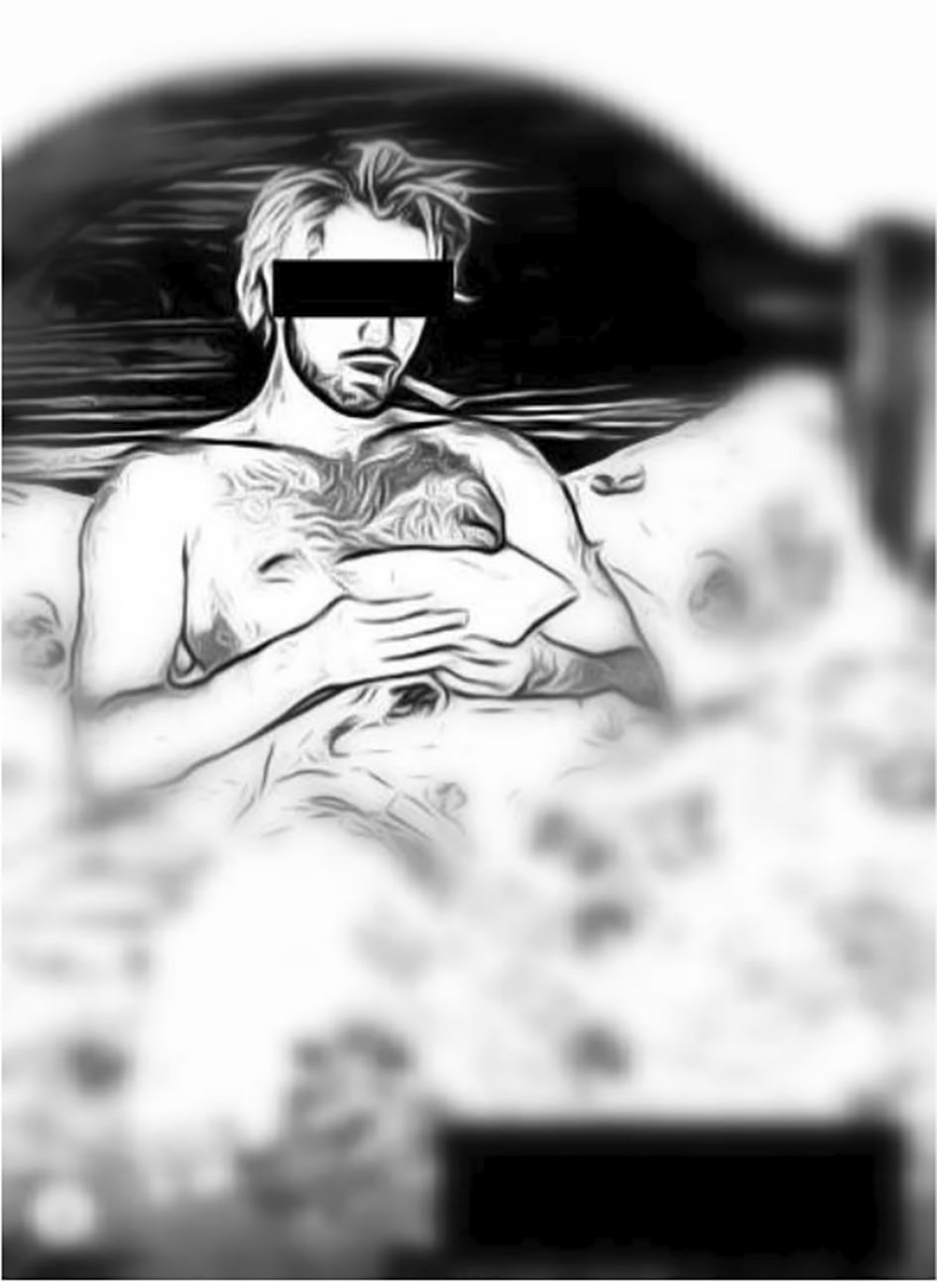
Description: Subversion of habitual self-presentation rarely occurred. Single exceptions included the overweight or subjects with children, or the use of irony to create distance from the medium and societal behavior regarding gender/dating stereotypes. In these pictures symbols or actions are presented but not exercised that in both cases address the stereotypical presumptions about the male provider role. In two cases the person is half naked, having a cigar or a cigarette in his mouth which is not lit, holding play money.

Meaning: These presentations stick out in number and logic in comparison to the other types. Here the subjects refer to the social expectations revealing either personal information about responsibilities, everyday life and restrictions, like having children or being handicapped or take a personal stand in the form of distancing the self from norms and gender-roles by using irony and exaggeration or challenging habitual aesthetics. Subjects thus invite viewers into a specific lifestyle, milieu or political stand and intellectual capability (humor) for a communicative relationship and positioning in a rather provocative/ polarizing way.

All types may occur with optional cross-sectional criteria like hats, different types of clothes, gestures and filters, such as color schemes. Filters and color schemes vary in intensity, from slight manipulations of the skin texture up to drastic blurs almost beyond recognition. These pictures come as overexposed or beauty filters are used, so that facial expressions and skin texture seem mask-like and artificial. A tension in picture editing between seemingly small edits, or artsy and explicit playing with graphical tools and drastically optimizing/editing occurs—for instance with snap chat filters, making the face cat-like or adding artificial graphical extras like birds, tattoos, freckles, and so forth.

New 2020 intersectional criterion: COVID-19 mask.


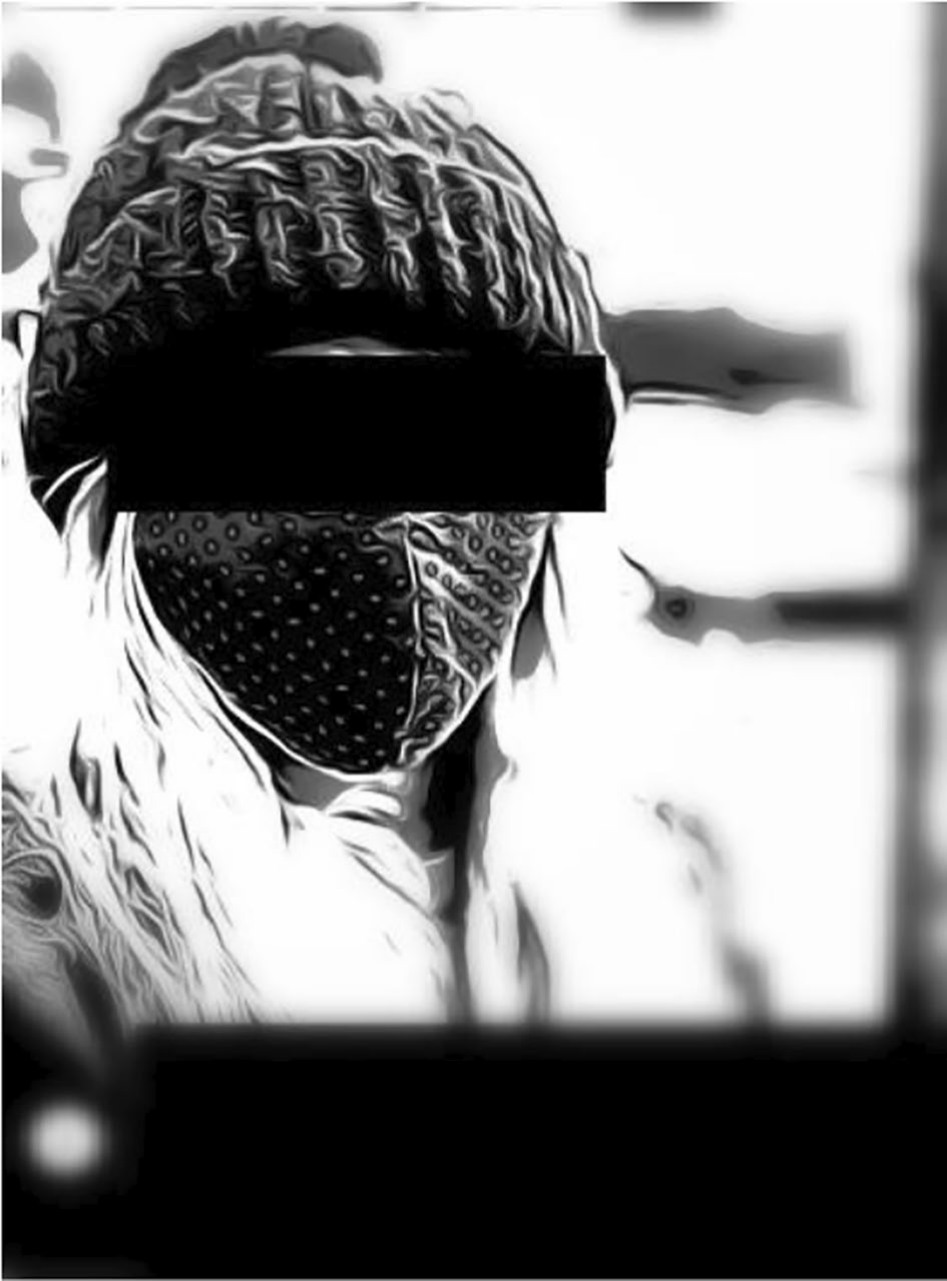
Given the situation of COVID-19, we were cautious to conclude about tendencies and comparison. A preliminary comparison shows a significant rise in selfies by up to almost 50%, many more sunglasses covering the face are presented (which probably correlates with seasons). Furthermore, there is a rise in pictures with accompanying subjects and there is no picture in the type of irony. There are similarly many pictures taken outside and inside, just as many incognito, alongside one new cross-sectional criteria: the mouth and nose (COVID-19) masks. This leads to speculation about whether the increase of selfies might be responsive to the pandemic and isolation or a collective tendency, which will be disclosed with further data series.

## Profile Pictures (in Mobile Online Dating Apps) at the Intersection Between Social Norms and Subjective Sovereignty

According to the logic of the reconstructive research paradigm, not everything is consciously enacted, but nothing is randomly created. Based on our results we can infer that subjects (visual) self-presentation in mobile online dating apps relies on implicit knowledge about social norms, unwritten rules and (assumed) expectations of an anonymous, imagined audience. These implicit rules, expectations and audiences apparently require highly controlled (selfie type) or staged (professional photoshoot type) representations, references to romantic ideals (informative type) or the playful nature of love (incognito type), the significance of authenticity (snapshot type) or one’s affability (sociability type).

As the vast majority of the profile pictures in our sample belongs to one of these types. Across almost all of them, we see rather similar pictures that comply with general viewing habits, showing subjects in habitual motifs. Considering online dating as a specific social arena, Tinder predominantly constitutes—at least visually—a sphere of pleasure and leisure, beyond conventional everyday life with its responsibilities or let alone challenging circumstances like having children or being handicapped.

The prescribed logic of self-presentation in online dating app profiles seems to reduce the uniqueness of the subject to a uniqueness of many. Subjects seem rather to strive for being easy to categorize and decide on within seconds—thus exercising restrictive agency in Holzkamp's (1983) sense—than presenting the self as different, unique or challenging. In the tension between commonness and uniqueness this complies to the rather quantitative logic of being likable by many and compliant to the other’s practices. Choosing one of few characters or roles investing in an optimized and compliant impression management (Goffman, 1959) might reflect that in the practice of swiping along conformity seems rewarded by getting likes and matches. One reason for this is possibly the perceived time pressure limiting the time for reflecting about the reasons for one’s decisions, where non-conformity could be disturbing and is rejected in an accelerated dynamic process. In consequence showing one’s uniqueness seems to compete with being likeable.

Conformity assumingly pays off also in terms of self-validation, with likes being the relevant currency; users strive for rewards by presenting themselves in certain successful ways – which then reduces the potential multitude of options to display oneself. This is reasonable as presenting the self on the app exposes a person to a liberal logic in Roses terms (2006). Failure then is counted as one’s own fault and personal shortcoming. Failing in this context might be rejection in the form of relatively few matches, getting unmatched, low answering rate or number of physical dates. These rejections then are attributed to not being either good enough at using the app, having a repelling profile or even worse to being not good enough as a person or at least not good enough in comparison to others (Gergen, 1991; Festinger, 1954). This again has, in Gergens understanding of the self, a negative impact on both self-esteem and self-worth.

In sum, inner psychological processes, such as validation and adjustment of the self, in this specific online-dating context is thus negotiated with imagined others, possibly idealized others creating a digital projective surface for supposedly social yet mostly projective self-processes.

Few users resist the omnipresent restrictive logic with pictures disrupting the common ways of self-presentation. These exceptions contain different qualities, firstly by suspending the subject, inviting into a space for interpretation and projection, directing the other into reflective processes about norms and desires. Second, by a counter-habitual self-presentation for instance by creating distance through reflection and irony Here, subjects undermine the logic of immediate attractiveness and easily categorizable self-presentation—for instance, a guy sitting on the toilet or a guy posing in bed with an unlit cigar while throwing monopoly money. These exceptions expose more than most of the other types, in terms of a reflexive and provocative action using their own embodiment and referencing social norms and the respective medium by exaggerating the stereotypical male role or using rather repelling motifs. The third quality of exception counts profiles including rather personal insights into having children (1 of 542), being overweight (2 of 542), being handicapped, having odd hobbies or specific sexual preferences or fetishes (3 of 542). Being resistive towards the social norms of appropriateness and attractiveness in an (online) dating context might be a sign of generalized agency (Holzkamp, 1983), for instance of overcoming restrictive social rules and thereby widening one’s scope of action. Although comparatively few pictures belong to these “resistive” types, they are nevertheless significant, since they show the actual possibilities of self-expression, which most people do not use.

## Ethics and Limitations

### Ethics

Using data in the form of screen grabbing in social networks should be considered in ethical terms. As Condie, Lean and Wilcockson (2017) argue, specific consideration is necessary when collecting data in social media. In the social media environment, data is accessible, which does not equal the right to re-use it without protecting personal rights, as data is assumed to always contain personal information (Evans et al., 2015). At the same time, research about digital realities/social media is valid and important. The established and published research practices range from simply screen grabbing and publishing content (Abidin, 2016: Instagram content) to paraphrasing texts so the account cannot be identified by an internet search. For this research design on images, we follow ethics in social media research based on considerations of the association of Internet research, Evans et al (2015) and Fossheim & Ingierd (2015). The research is first, not violating the Tinder terms and conditions, which mainly prevent commercialization and re-use of pictures or content for commercials. Second, we did not engage in a relationship with the account holders and we secured the privacy of the data by following the data protection declaration from our university, keeping it strictly confidential. Finally, the pictures presented in the typology are anonymized to a degree that even through a google picture search neither the account, nor the account holder’s digital identity can be revealed, or the person recognized.

### Limitations

There are some limitations and specifications to be mentioned for this contribution. The study is relying on data collected on one specific application, namely Tinder; it has yet to be researched whether the logic is generalizable for other mobile online dating applications or if it is Tinder specific. Other applications, like Bumble, discern in both digital architecture, marketing, and the social group of typical users as target group. Furthermore, both series are collected in northern Europe, further research might show to what degree national characteristics play a role. Although one might assume that there are minor differences in the global west, yet there are differing beauty standards and cultural differences. For instance, regarding compliant self-presentation, optimization (e.g., plastic surgery playing different cultural roles in inter alia China, Brazil, US context, and so forth: discussed in Sturm-O’Brien et al., 2010) and many more.

In the conduct of research, we collected data taking the role of gender into account, presuming differences, aiming for a balanced sample. In the analysis, the presumptions did not persist in great manner. There are only a few gender differences, many types are gender overarching, for instance the types selfie, informative, and incognito. Gender differences shown in former research for instance regarding sport equipment did not present great relevance for the findings.

This aspect could be focused with regards to simulation effects, where users simply mirror the other’s self-presentation. Users imitate the pictures they see. As the pictures the users see—at least in a heterosexual setting—come from the opposite sex, male and female presentations would align.

When interpreting the findings there are algorithm related aspects to consider: users can choose to let Tinder select their most successful picture out of the possible up to nine profile pictures. Nevertheless, this does not change the significance of the profile pictures, as the algorithm can still only choose from the users’ portfolio. Yet, this aspect could have a streamlining effect as it accelerates the conditioning of specific self-presentation. The reconstructive serial analysis is focused and limited to the intersubjective orientation that again functions as a storyline for behavior—the inherent logic and social rules—it cannot and does not aim for a reconstruction of the individual expectation, motives, or aspired impression/intent nor does it contribute to the individual perception of these forms of self-presentation.

Finally, building a typology contains paradigmatic limitations that should be considered when interpreting the results. When building typologies, the researcher is biased to search for minimum and maximum contrast, resulting in typicality of self-presentation; this does not imply that there is not individuality in the self-presentation and individual nuances.

## Conclusion and Outlook: Coming to an Understanding of Selves When Mobile Online Dating

Serial picture analysis in the predominantly visual context of mobile online dating gives valuable insights into implicit social norms and respective subjective referencing in the presentations of the selves, beyond the seemingly obvious. The results show that these norms are oriented towards easily categorizable and countable common motives and presentations, indicating a strong restrictive context and collectively complied norms. The great majority of users orient the self-presentation strongly on these social norms and do not exhaust the vast possibilities of variety, presenting contrary motives or create unique and individual profiles. This goes far beyond the discourse on self-presentation in mobile online dating where picture analysis so far mostly focuses on quantitative analysis of presented motives, colors, and objects and mere speculations about users’ motifs. This contributes by a focus on the actual pictures following the reconstructive logic serving with insights to the users’ implicit knowledge—users know more than they know to know—and can thus reveal more than reflections can access, for instance by asking users about intentions and (post-constructed) reasoning for their self-presentation. The findings add to the current body of research by highlighting a paradox: while the vast majority of users are looking for special moments, for (the) big love, and want to be perceived as someone special, these desires are counteracted by their own self-presentations. This self-assignment to a few types corresponds to an accelerated practice of constant wiping, the result of which—many similar and thus also boring options—they often complain about but produce themselves.

These insights lead to several general questions in the context of social practices and specifically relationship formations. We suggest that subjective behavior is not determined or one-sided causally shaped by the application (Thornham & Cruz, 2015). Instead, we assume that digital realities and the established practices have two-sided impacts wherein subjects and technical context reciprocally shape, acquire, and grow with each other (Schraube, 2009). One of peoples’ main motives of using mobile online dating applications is finding love and a partner and creating special moments (Degen & Kleeberg-Niepage, 2020; Timmermans & DeCaluwe, 2017). The analysis directs into a deeper understanding of a tension between motifs of presenting the self to find a fitting partner and to be understood as special and the strive for validation and thus compliant and self-presentation conformity. The established usage of the application seems to establish an intersubjective understanding and increase of a viewing habit conform presentation of the self, possibly due to the accelerated usage of swiping and the orientation towards a quantitative logic that possibly undermines individuality in the presentation. This contributes to the research of online dating by analyzing the actual self-presentation enriching the understanding in the sphere between motives, (high) hopes and expectations and the actual behavior. The practice seems immediately contradictory to the original aim of finding a fitting match. What does the practice of common and somehow unspecific self-presentation mean for user’s ideas of intimate relationships? How does such a self-presentation relate to their self-concept and what does it mean to them personally?

Further research will focus on detailed analyses of single images from our typology on how individual self-presentations are created, leading to a deeper understanding of the presumed significance of age, milieu, class, or gender within these processes.
